# Novel Spotted Fever Group Rickettsiosis, Brazil

**DOI:** 10.3201/eid1603.091338

**Published:** 2010-03

**Authors:** Mariana G. Spolidorio, Marcelo B. Labruna, Elenice Mantovani, Paulo E. Brandão, Leonardo J. Richtzenhain, Natalino H. Yoshinari

**Affiliations:** University of São Paulo, São Paulo, Brazil

**Keywords:** Rickettsiosis, spotted fever group, Rickettsia, bacteria, infection, Brazil, dispatch

## Abstract

We report a clinical case of spotted fever group rickettsiosis acquired in São Paulo, Brazil. Definitive diagnosis was supported by seroconversion between acute-phase and convalescent-phase serum samples. Molecular analysis of skin samples indicated the agent was a novel spotted fever group strain closely related to *Rickettsia africae, R. parkeri,* and *R. sibirica*.

*Rickettsia rickettsii* is the etiologic agent of Rocky Mountain spotted fever (RMSF). During the past 2 decades, a clear reemergence of RMSF has been seen in southeastern Brazil, where ≈350 laboratory-confirmed cases (case fatality rate ≈30%) have been reported (*1*). Most of these cases were confirmed solely by serologic-based techniques; specific identification of the *Rickettsia* species was not achieved. However, because these cases were clinically and epidemiologically compatible with RMSF, the agent was presumed to be *R. rickettsii* (*1*).

The occurrence of *R. parkeri* in Brazil has been restricted to ticks; human clinical infection has been reported in the United States, and possibly in Uruguay (*1*). Additionally, a few clinical cases caused by *R. felis* or *R. typhi* have been reported in southeastern Brazil during the 21st century (*2**,**3*). We report a clinical case of SFG rickettsiosis in a patient from southeastern Brazil. Molecular analysis of clinical samples showed that the patient was infected by a novel SFG strain.

## Case Report

**Figure 1:**
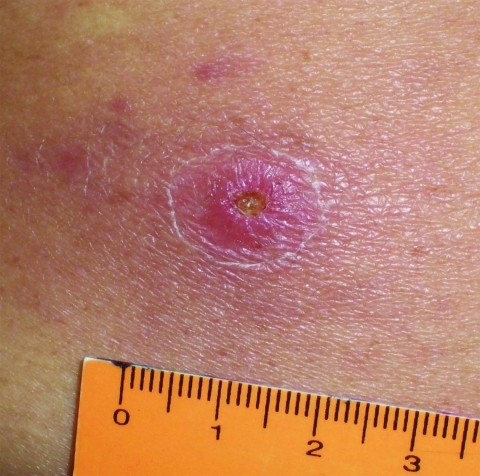
Inoculation eschar on the lumbar region of the back of a patient infected with a *Rickettsia* sp. from the Atlantic rainforest in the state of São Paulo, southeastern Brazil.

On May 2, 2009, a 66-year-old man was bitten by a tick on his lumbar region while walking on his ranch within an Atlantic rainforest area. Although his primary residence was in the urban area of Santo André within the São Paulo Metropolitan region (where he reported never having been bitten by ticks), he often visited his ranch in Barra do Una, a village within the Peruíbe Municipality, southern coastal region of the state of São Paulo (where he reported having been bitten by ticks several times). The area is within a large Atlantic rainforest reserve and is <50 m above sea level. The patient reported no travel to additional locations during the previous 3 months. Ten days after the tick bite (May 12, 2009), the patient reported the first episode of fever (≈39°C) and took acetaminophen. On May 15, 2009, he visited a doctor, who prescribed oral cephalexin (500 mg, 6×/6 h). On the next day, a macular rash appeared on his arms and legs, associated with muscle and joint pain. Fever was still present (39.5°C). On May 19, 2009, the patient had continuing fever (39.5°C), a macular rash (without itch) on his arms and legs, arthralgia, and myalgia on lower regions of the arms and legs and hands. The patient had an eschar on the lumbar region (Figure 1), exactly where he had removed an attached tick on May 2, 2009. He admitted that the tick remained attached to his skin for at least 20 hours until being removed and discarded.

Based on suspicion of rickettsial disease, blood samples were collected the same day, and doxycycline (100 mg, 12×/12 h) was prescribed for 10 days. Three days later (May 22, 2009), the patient returned to the laboratory where a new blood sample was collected, and a skin biopsy of the eschar was aseptically performed. The patient had not had a fever since May 20, 2009 (1 day after initiation of doxycycline therapy), but still had macular rash and joint and muscle pain. A third blood sample was collected 13 days later (June 4, 2009), and no clinical abnormalities were found.

Blood serum was tested by using an immunofluorescent antibody assay with antigens from 6 *Rickettsia* species that are present in Brazil: *R. rickettsii*, *R. parkeri, R. felis, R. amblyommii, R. rhipicephali,* and *R. bellii* (*4**,**5*). Serum samples were tested with a goat antihuman immunoglobulin (Ig) G or a goat antihuman IgM fluorescein isothiocyanate conjugate (Sigma Diagnostics, St. Louis, MO, USA). Patient showed seroconversion with a minimum 8× increase in titers of antibodies against *Rickettsia* spp. between the first samples (collected during the febrile period) and the third blood sample (collected 16 days later) (Table).

**Table:** *Rickettsia* spp. serologic titers by immunofluorescent antibody assay for a Brazilian patient in the state of São Paulo, Brazil, 2009*

Antigen	Titers							
	May 19			May 22			Jun 4	
	IgM	IgG		IgM	IgG		IgM	IgG
*Rickettsia rickettsii*	64	64		128	256		512	512
*R. parkeri*	<64	<64		256	256		1,024	512
*R. felis*	<64	<64		<64	128		<64	128
*R. amblyommii*	<64	<64		128	<64		512	512
*R. rhipicephali*	<64	64		128	256		512	512
*R. bellii*	64	<64		256	128		512	512

*Ig, immunoglobulin.

DNA was extracted from the skin biopsy specimen by using the DNeasy Blood and Tissue Kit (QIAGEN, Hilden, Germany) according to the manufacturer’s instructions and tested by a battery of PCRs to amplify fragments of the rickettsial genes citrate synthase (*gltA*) (primers CS-78, CS-323, CS-239, CS-1069), outer membrane protein (*ompB*) (primers 120-M59, 120–807), and *ompA* (primers *Rr*190.70p, *Rr*190.602n), as described (*6*). PCR products were purified and sequenced (*4*). Partial sequences were subjected to BLAST analysis (*7*) to determine similarities to other *Rickettsia* species. Partial *gltA* sequence (1,078 bp) showed 100% similarity to *R. sibirica* (RSU59734), 99.9% to *R. parkeri* (EF102236), and 99.8% to *R. africae* strain S (RSU59735). Partial *ompB* sequence (740 bp) showed 99.2% similarity to *R. africae* (AF123706) and *R. parkeri* strain NOD (EU567179), and 98.6% to *R. parkeri* (AF123717) and *R. sibirica* (AF123726). Partial *ompA* sequence (463 bp) showed 99.8% similarity to *R. africae* strain S (RSU43805), 99.6% to *R. africae* (EU622980), 99.1% to *R. sibirica* (AF179365), and 98.3% to *R. parkeri* (RPU43802).

**Figure 2:**
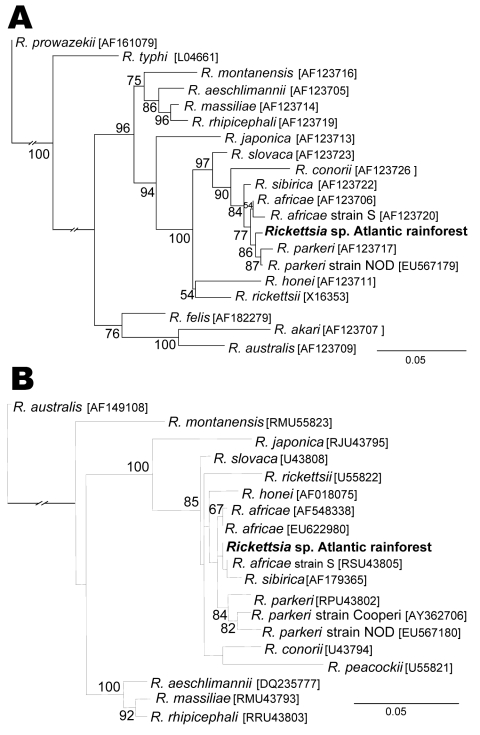
Molecular phylogenetic analysis of *Rickettsia* sp. strain Atlantic rainforest detected in a patient from the State of São Paulo, Brazil. A) A total of 740 unambiguously aligned nucleotide sites of the rickettsial outer membrane protein (*ompB*) gene were subjected to analysis. B) A total of 463 unambiguously aligned nucleotide sites of the rickettsial *ompA* gene were subjected to analysis. Bootstrap values >50% are shown at the nodes. Numbers in brackets are GenBank accession numbers. The strain isolated in this study is indicated in **boldface**. Scale bars indicate nucleotide substitutions per site.

For each rickettsial gene, partial sequences were aligned with the corresponding sequences of other *Rickettsia* species available in GenBank, and rooted phylogenetic trees were built with PAUP 4.0b10 (*8*) by using the maximum likelihood method with an heuristic algorithm and the transition model + the Γ, transversion model + Γ, and the general time reversible + Γ + proportion invariant model for *gltA, ompB,* and *ompA,* respectively, as determined by Model Test (*9*). Tree stability was assessed by bootstrapping >1,000 replicates. In all trees, the sequence from the Brazilian patient, designated as *Rickettsia* sp. Atlantic rainforest, grouped in a cluster composed by different strains of *R. africae, R. parkeri,* and *R. sibirica.* This cluster was supported by high bootstrap value for *ompB* tree, but low for the *ompA* tree (Figure 2). Little divergence was observed between SFG species in the *gltA* tree; clusters were generally supported by low bootstrap values (data not shown). Partial sequences (*gltA, ompB, ompA*) from *Rickettsia* sp. strain Atlantic rainforest generated in this study were deposited into GenBank and assigned nucleotide accession nos. GQ855235–GQ855237, respectively.

## Conclusions

We report a clinical case of SFG rickettsiosis acquired in an Atlantic rainforest area of the state of São Paulo, Brazil. Definitive diagnosis is supported by demonstrating a minimum 8× increase in titers between acute-phase and convalescent-phase serum samples, and by identification of rickettsiae in an acute-phase tissue sample (eschar), which was confirmed as a novel SFG strain and designated as *Rickettsia* sp. strain Atlantic rainforest. Genetic analyses indicated that this new strain was similar to *R. africae, R. parkeri,* and *R. sibirica.* The clinical profile of the Brazilian patient was similar to the disease caused by these 3 rickettsial species in the United States (*R. parkeri*) and in the Old World (continents of Asia, Europe, and Africa [*R. africae* or *R*. *sibirica*]), that is, mild fever, muscle and joint pain, eschar, rash, and no deaths (*10**–**12*). We did not observe regional lymphadenopathy, a clinical sign usually associated with *R. parkeri, R. africae,* and *R. sibirica* (*10**–**12*) infection, possibly because the inoculation eschar was on the lumbar region of the back.

It was recently proposed that a new *Rickettsia* species should not show >99.9%, 99.2%, and 98.8% similarity for the *gltA*, *ompB*, and *ompA* genes, respectively, with the most homologous validated species (*13*). The strain detected in the Brazilian patient showed similarity values equal to or greater than the above threshold values for >2 genes of either *R. africae* or *R. parkeri* or *R. sibirica.* Thus, we cannot identify the species for *Rickettsia* sp. strain Atlantic rainforest. Notably, it has been proposed that closely related species, such as *R. parkeri* and *R. africae*, should be considered strains of 1 species (*12*).

## References

[1] Labruna MB. Ecology of *Rickettsia* in South America. Ann N Y Acad Sci. 2009;1166:156–66. 10.1111/j.1749-6632.2009.04516.x 19538276

[2] Raoult D, La Scola B, Enea M, Fournier PE, Roux V, Fenollar F, A flea-associated *Rickettsia* pathogenic for humans. Emerg Infect Dis. 2001;7:73–81. 10.3201/eid0701.010112 11266297 PMC2631683

[3] Silva LJ, Papaiordanou PMO. Tifo murino (endêmico) no Brasil: relato de caso e revisão. Rev Inst Med Trop Sao Paulo. 2004;46:283–5. 10.1590/S0036-46652004000500010 15517036

[4] Horta MC, Labruna MB, Pinter A, Linardi PM, Schumaker TTS. *Rickettsia* infection in five areas of the state of São Paulo. Mem Inst Oswaldo Cruz. 2007;102:793–801. 10.1590/S0074-02762007000700003 18094887

[5] Labruna MB, Horta MC, Aguiar DM, Cavalcante GT, Pinter A, Gennari SM, Prevalence of *Rickettsia* infection in dogs from the urban and rural areas of Monte Negro Municipality, western Amazon, Brazil. Vector Borne Zoonotic Dis. 2007;7:249–55. 10.1089/vbz.2006.0621 17627445

[6] Guedes E, Leite RC, Prata MCA, Pacheco RC, Walker DH, Labruna MB. Detection of *Rickettsia rickettsii* in the tick *Amblyomma cajennense* in a new Brazilian spotted fever–endemic area in the state of Minas Gerais. Mem Inst Oswaldo Cruz. 2005;100:841–5. 10.1590/S0074-02762005000800004 16444414

[7] Altschul SF, Gish W, Miller W, Myers EW, Lipman DJ. Basic local alignment search tool. J Mol Biol. 1990;215:403–10. 2231712 10.1016/S0022-2836(05)80360-2

[8] Swofford DL. PAUP*. Phylogenetic analysis using parsimony (*and Other Methods). Version 4. Sunderland (MA): Sinauer Associates; 2000.

[9] Posada D, Crandall KA. Modeltest: testing the model of DNA substitution. Bioinformatics. 1998;14:817–8. 10.1093/bioinformatics/14.9.817 9918953

[10] Parola P, Paddock CD, Raoult D. Tick-borne rickettsioses around the world: emerging diseases challenging old concepts. Clin Microbiol Rev. 2005;18:719–56. 10.1128/CMR.18.4.719-756.2005 16223955 PMC1265907

[11] Paddock CD, Finley RW, Wright CS, Robinson HN, Schrodt BJ, Lane CC, *Rickettsia parkeri* rickettsiosis and its clinical distinction from Rocky Mountain spotted fever. Clin Infect Dis. 2008;47:1188–96. 10.1086/592254 18808353

[12] Walker DH, Ismail N. Emerging and re-emerging rickettsioses: endothelial cell infection and early disease events. Nat Rev Microbiol. 2008;6:375–86. 10.1038/nrmicro1866 18414502

[13] Fournier PE, Raoult D. Current knowledge on phylogeny and taxonomy of *Rickettsia* spp. Ann N Y Acad Sci. 2009;1166:1–11. 10.1111/j.1749-6632.2009.04528.x 19538259

